# Hybrid Swarm Intelligence Optimization Approach for Optimal Data Storage Position Identification in Wireless Sensor Networks

**DOI:** 10.1155/2015/597486

**Published:** 2015-02-04

**Authors:** Ranganathan Mohanasundaram, Pappampalayam Sanmugam Periasamy

**Affiliations:** ^1^School of Computing Science and Engineering, VIT University, Vellore 632014, India; ^2^Department of ECE, K.S.R. College of Engineering, Tiruchengode 637215, India

## Abstract

The current high profile debate with regard to data storage and its growth have become strategic task in the world of networking. It mainly depends on the sensor nodes called producers, base stations, and also the consumers (users and sensor nodes) to retrieve and use the data. The main concern dealt here is to find an optimal data storage position in wireless sensor networks. The works that have been carried out earlier did not utilize swarm intelligence based optimization approaches to find the optimal data storage positions. To achieve this goal, an efficient swam intelligence approach is used to choose suitable positions for a storage node. Thus, hybrid particle swarm optimization algorithm has been used to find the suitable positions for storage nodes while the total energy cost of data transmission is minimized. Clustering-based distributed data storage is utilized to solve clustering problem using fuzzy-C-means algorithm. This research work also considers the data rates and locations of multiple producers and consumers to find optimal data storage positions. The algorithm is implemented in a network simulator and the experimental results show that the proposed clustering and swarm intelligence based ODS strategy is more effective than the earlier approaches.

## 1. Introduction

The approach here in wireless sensor network (WSN) with regard to data storage approach aims at identifying the best data storage positions which basically is the primary issue and which encompasses many challenges. Inappropriate approaches may lead to significant energy loss, increased communication overheads, and a shortened sensor network lifetime as described in [1, 2]. On the other hand an appropriate data storage approach can efficiently minimize delays occurring in processing queries and energy consumed and additionally also lengthen the lifespan of the sensor network as mentioned in [3]. Therefore, apt and well-suited approaches that are inherently efficient are imperative to adjust data position which further can help minimize costs of storage and enable identifying query as mentioned in [4].

Producers as well as consumers rate of data and respective distances from the path leading to storage node are two essential aspects influencing data storage-related communication cost. The data rate of producers represents the data producing rate from producers. The data rate of consumers represents the data querying rate from consumers. The data rate generally does not alter in a fixed application-specific time interval. For instance, where there are only one producer and one consumer, data would be stored closer to the consumer rather than to the producer, when the query rate is higher than the data producing rate, and vice versa. In a practical sensor network, the storage node is closer to the producers and consumers; the cheaper one is to store and query a fixed quantity of data. An effective formulation would be to place data adaptively based on network state so that the communication cost is reduced once the data storage position is fixed which can be found in [5].

These two issues have already been discussed with tree structure based sensor network but, in the tree structure, the data rates of producers and the query rate of the base station are predictable and the optimization of the cost would be easy as data storage placement is based on the data volumes which can be found in [6]. But, in a mesh network topology, there are multiple producers and consumers and everyone is looking to exploit a particular event at the same time. Some of the previous works have dealt with the geographical locations of producers and consumers but the problem of the data rates was not taken into consideration which can be found in [7]. It is a fact that if the network topology is fixed, the storage node position is also fixed and adaptive storage principles are not required to minimize energy consumption in sensor networks. But the limitation is that the storage node position is neither location aware nor adaptive to network state.

Recently, [8] presented an approach which focused on the single-node storage problem in a wireless sensor network with a mesh topology, where information collected from all producers is sent to a storage node and all consumers retrieve information from there. But data load is usually asymmetric in a mesh network topology which can be found in [9]. This unbalanced data volume would result in an uneven energy consumption distribution between different sensor nodes which may result in narrow network lifetime with better energy consumption. Thus, [8] presented an optimal data storage (ODS) strategy in a WSN that facilitates the storage location to change dynamically, with respect to the geographical locations of both producers and consumers and the data rates at which data are being exchanged. This approach is observed to minimize the energy consumption of the entire network. Thus, storage position changes dynamically in response to data rates of nodes and their geographical locations. But the main limitation of this work is the utilization of ODS and near-optimal solution.

The most significant issue in the data storage approach is to gain suitable positions for a limited number of storage nodes in all nodes to make energy efficient, thus extending the lifespan of all wireless sensor networks. So, this research work extends the work which can be found in [8] through the utilization of metaheuristic swarm intelligence optimization algorithm.

In this paper, storage node position problem is considered and, hence, hybrid particle swarm optimization (PSO) is used to gain the suitable positions for *k* storage nodes in WSN based on the energy cost of data transmission. Reducing data access energy consumption here has been made possible through the presentation of an adaptive clustering based on data storage (CBDS) algorithm while employing FCM clustering. CBDS has appropriately adapted adjusted location of data storage through the process of calculation of data storage and query access costs, respectively.

## 2. Model and Problem

### 2.1. Network Model

**Figure 1:**
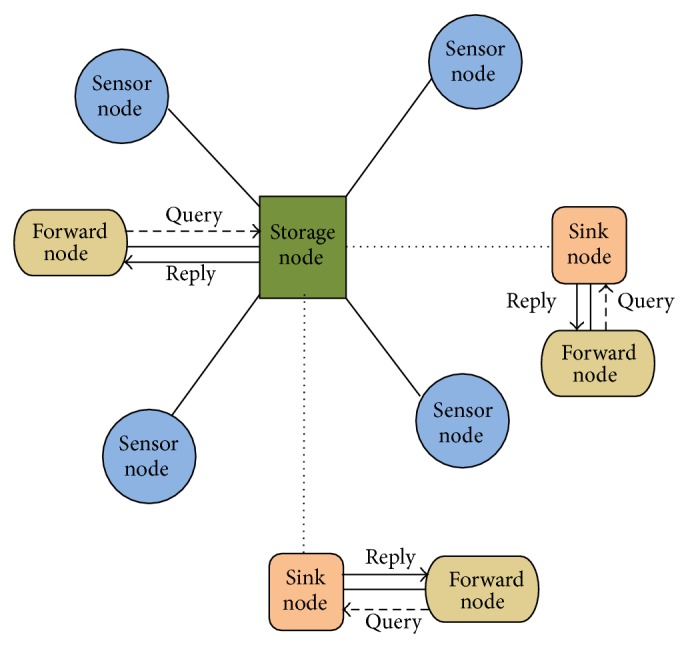
Wireless sensor network model.

A two-tier data storage approach for wireless sensor networks is presented in this paper, which comprises of three types of nodes.  Figure 1 gives the corresponding network model. Sensor node gains the sensing data and sends it to the neighboring storage nodes or sink node which can be found in [10]. Sink node allows the queries of user, disperses them to all storage nodes, and aggregates all replies.

There are three types of nodes as shown in Figure 1 in data storage approach, which is defined as follows and can be found in [10].

- Sink node: there is merely one sink node which may accept request of the user and precedes the suitable reply.
- Storage node: a storage node receives unrefined data gathered by the near sensor nodes and stores them. When sink node spreads out query message, storage node provides a reply by the unrefined data stored in it. Storage node also gathers the sensing data nearer to the surroundings and forwards query message and reply.
- Sensor node: sensor node gains sensing data and sends them, and it also forwards the unrefined data collected by nearer sensor nodes to storage node or other sensor nodes.
- Forward node (FN): these nodes always forward the data towards the sink through a routing path. The outgoing data is safely sustained and forwarded until the data reaches the storage node.

**Figure 2:**
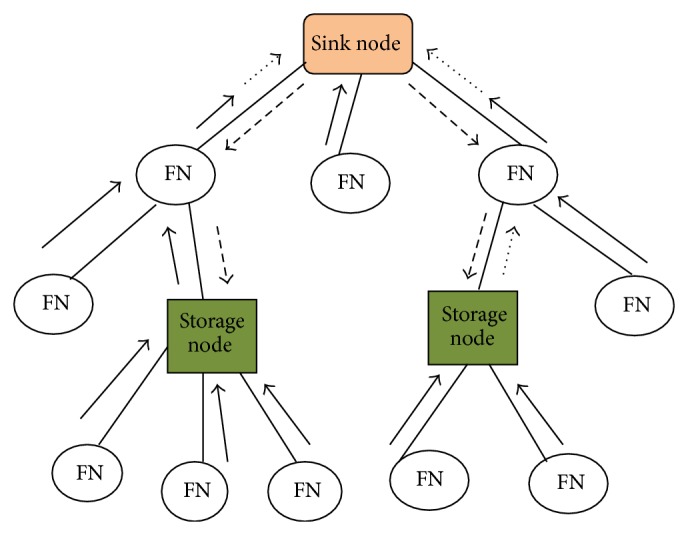
An example of data storage in wireless sensor networks.

An example of data storage in WSN is clearly depicted in Figure 2.

### 2.2. Problem Formulation

**Figure 3:**
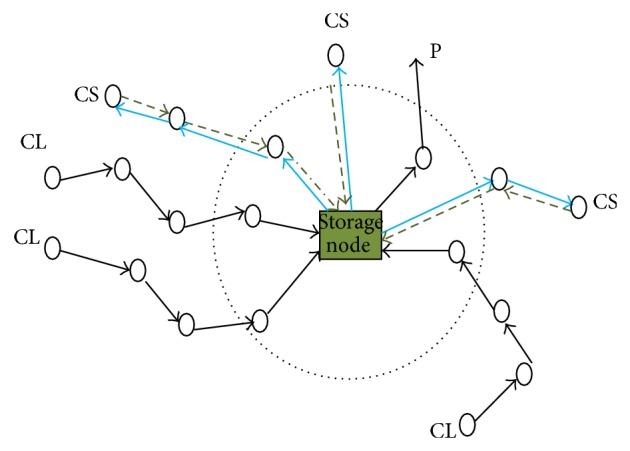
Data storage in wireless sensor networks, a scenario.

The present research work focuses on the adaptive data storage issue through the data storage process and implicated communication overheads. The optimal data storage approach based on hybrid swarm intelligence optimization problem to find the storage node position with the minimum cost is proposed in this approach.  Figure 3 shows a circumstance in which collectors (CLs) identify events and accumulate critical data into a storage node. Consumers (CSs) then issue queries to the storage node for obtaining applicable information.

The communication overheads in each one of these three types are represented as storage cost or *C*_Storage_, diffusion cost or *C*_Diffusion_, and reply cost or *C*_Reply_. The diffusion cost and reply cost can be incorporated as query cost or *C*_Query_; that is, *C*_Query_ = *C*_Diffusion_ + *C*_Reply_. The communication overheads are subjective by the transmission routes as recognized by the sites of producers and consumers. A number of suppositions have been considered for forming the data storage as discussed in [11] as follows.

- All nodes apart from the base station are equal, whereas *R* is referred to be a radio transmissions range for all nodes. There are *N* nodes in the network and they are marked as 1,2,…, *i*,…, *N*. Let *i* be a node in the network and its location is referred to as (*x*_*i*_; *y*_*i*_).
- The base station has the structural information of the network by monitoring and gathering the network information and can carry on with complex calculation. It can calculate the optimal storage position as asked for in the many-to-many model.
- A communication edge *e*_*ij*_ is present among any pair of nodes *i* and *j* that are in radio range. To transmit *s* data units beside the edge, the energy cost of the sender and receiver is *σ*_tr⁡_ + *δ*_tr⁡_ *s* and *σ*_re_ + *δ*_re_ *s* correspondingly [11], where *σ*_tr⁡_ and *σ*_re_ are startup energy costs for transmitting and receiving a data packet, respectively, and *δ*_tr⁡_ and *δ*_re_ are energy cost for transmitting and receiving a data unit (byte), respectively. Compared with the energy cost for data transmission, the computation energy cost can be unseen. In this paper, only the energy cost in data transmission is taken into consideration.
- An event takes place arbitrarily at any place at any time. A node *i* sends event data to or gathers data from a storage node *k* at the fixed rate *R*(*i*) in a unit time interval.
- In a common case, there are *m* producers and *n* consumers connected to an event in the network concurrently (1 ≤ *m*, *n* ≤ *N*  and  *m* + *n* < *N*) and the storage node is node *k*. Let *m* producers be *p*_1_, *p*_2_,…, *p*_*m*_ and let *n* consumers be *c*_1_, *c*_2_,…, *c*_*n*_.  *C*_stroage_(*i*, *k*) represents the storage cost when producer *i* sends event data to node *k*, and *C*_query_(*j*, *k*) represents the query cost when consumer *j* diffuses a query request to node *k* and attains the appropriate data.

### 2.3. Energy Cost Formulation

Consider a database *D* to be microdata table that stores the confidential information about a set. The main goal of this work is to find out the large amount energy-efficient storage position, that is, the position where the energy consumption linked with data storage, query distribution, and result reply is negligible. A statistical model is formed to enumerate, from all feasible storage positions, the cost of storage and query and point out the minimal energy cost min⁡{*C*_storage_ + *C*_query_} in the optimal storage policy.

The storage cost *C*_storage_(*k*) is defined as a unit time interval which means sum of all costs of sending data to node *k* for storage from all producers, whereas, the query cost *C*_query_(*k*) is the sum of all costs of querying and gets hold of the relevant data commencing all consumers:

(1) $$\begin{array}{c} C_{\text{storage}}\left(k\right)=\overset{p_{m}}{\underset{i=p_{1}}{\sum }}C_{\text{storage}}\left(i,k\right),\\ C_{\text{query}}\left(k\right)=\overset{n}{\underset{i=c_{1}}{\sum }}C_{\text{query}}\left(i,k\right). \end{array}$$

The storage cost and query costs depend on both the data rate and the hop distance in the data transmission path. The data rate is significant to energy consumption of data storage and admission; the smaller the data rate is, the less it is to store and query a fixed quantity of data. To the storage node *k*, the following equation is used:

(2) $$\begin{array}{c} C_{\text{storage}}\left(i,k\right)=R\left(i\right)*h\left(i,k\right),\\ C_{\text{query}}\left(j,k\right)=R\left(j\right)*h\left(j,k\right), \end{array}$$

where *h*(*i*, *k*) and *h*(*j*, *k*) are the hop counts between the pair nodes (*i*, *k*) and (*j*, *k*), respectively; the total cost *C*_total_(*k*) is the sum of *C*_storage_(*k*) and *C*_query_(*k*) which is related to producers storing the data at node *k* and consumers getting back the suitable data from there:

(3) $$\begin{array}{c} C_{\text{total}}\left(k\right)=\overset{p_{m}}{\underset{i=p_{1}}{\sum }}R\left(i\right)*h\left(i,k\right)+\overset{c_{m}}{\underset{{i=c}_{1}}{\sum }}R\left(j\right)*h\left(j,k\right). \end{array}$$

Finding the optimal storage node, that is, to acquire minimal *C*_total_(*k*), is a complex problem because several factors can impact the storage position selection, for example, data rates and multiple paths between two arbitrary nodes in the network and the network structure. Though there may be multiple paths between two nodes, data are transmitted beside a fixed path for definite application-specific time duration if the network does not change radically because the path between any two nodes is firmed by the application's routing protocol.

The optimal storage position can be governed in the one-to-one model or many-to-many model in a sensor network based on the number of producers and consumers. In the one-to-one model, there are merely one producer and one consumer. On the other hand, the many-to-many model allows the survival of multiple producers and multiple consumers.

This work concentrates on attaining the optimal data storage position for one-to-one model in a mesh network topology. It is noted that the communication overhead is proportional to the size of message and the distance between producer and consumer, and the distance does not refer to the real distance but to hop counts in wireless sensor networks.

The total energy cost given in (3) is solved using a metaheuristic optimization algorithm called the particle swarm optimization (PSO).

The objective function of this approach would be to minimize the THD of the model:

(4) $$\begin{array}{c} \text{Minimize}f\left(x\right)=C_{\text{total}}\left(k\right). \end{array}$$

The constraints used in the approach are equality constraints and inequality constraints. The parameter which has to be optimized in this approach is *f*(*k*). The subject of limits considered in this research work is

(5) $$\begin{array}{c} {C_{\text{storage}}(k)}_{\min}<C_{\text{storage}}\left(k\right)<{C_{\text{storage}}\left(k\right)}_{\max},\\ {C_{\text{query}}(k)}_{\min}<C_{\text{query}}\left(k\right)<{C_{\text{query}}\left(k\right)}_{\max}. \end{array}$$

The optimal storage position can be obtained through one-to-one model and many-to-many model in a sensor network based on the number of CLs and CSs. In the one-to-one model, there are only one CL and one CS. However, the many-to-many model allows the existence of multiple CLs and multiple CSs. The optimal data storage position can be analyzed in linear topology. It is observed that the communication overhead is proportional to the message size and the distance between CLs and CSs, and the distance is based on the hop counts in wireless sensor networks.

## 3. ODS Algorithm

In this section, the optimal data storage (ODS) algorithm is introduced in one-to-one model (Figure 2) to find out the optimal storage node *k*. There are only one producer *i* and one consumer *j* to agent information in the network. Because the network is connected, there should be existence of the shortest path *P* that connects producer and consumer through the storage node *k*. The length of the path is calculated by the hop count between nodes *i* and *j*. Consider that the length of *P* is *L* and the distance among producer *i* and node *k* is a variable *x*. Therefore, the distance between consumer *j* and node *k* is *L* − *x*. By (5), the total energy consumption selecting *k* as the storage node is

(6) CTotalk=Ri∗hi,k+Rj∗hj,k=Ri∗X+Rj∗L−X=Rj∗L+Ri−Rj∗X.

From (6), an optimal storage location is obliged to minimize *C*_Total_(*k*).

### 3.1. Optimal Data Storage in Many-to-Many Model

In this section, we present the cost of data storage and query dealing out in a many-to-many model for linear, grid, and mesh network structures. Given *m* producers and *n* consumers, initially ODS algorithm is introduced to the linear, grid, and mesh network topologies. To resourcefully get hold of a storage location, near-optimal data storage (NDS) algorithm is proposed in the mesh network topology. It tells that, intended for a producer or a consumer, its part to the total cost only corresponds to its data rate and hop count to the storage node. Therefore, it is not necessary to differentiate producers from consumers.

### 3.2. ODS in a Linear Network Topology

Initially examine into the total cost of implementing optimal data storage in a linear sensor network that comprises of a group of sensor nodes placed beside a long and narrow area. Each producer gathers the sampled data in its sensing range and relays data towards a storage node. Each consumer gets hold of the suitable data from that storage node. For ease, each node relays data for nodes further away; that is, node *i* also relays the data collected by nodes 1 to *i* − 1 to the storage node and does not proceed with data aggregation.

Typical applications contain traffic monitoring and border control. Since all sensor nodes are placed in a line, then their coordinates are used to calculate the hop distance. Let *x*_*i*_, *x*_*j*_, and *x*_*k*_ be the coordinate of producer *i*, consumer *j*, and storage node *k*, respectively. As a result, *h*(*i*, *k*) and *h*(*j*, *k*) can be calculated as follows:

(7) $$\begin{array}{c} h\left(i,k\right)=\left|x_{i}-x_{k}\right|,\\ h\left(j,k\right)=\left|x_{j}-x_{k}\right|. \end{array}$$

At present, provide the optimal storage position in a linear network topology based on multiple producers and multiple consumers. For a while, scan the linear network from two ends just before the midst at the same time. A node with 0 data rate does not contribute several storage cost. Hence, when the data reaches a node whose data rate is zero, then just skip it and move further on. Because producers and consumers are treated uniformly and just their data processing rates count for the total storage cost, then select the final storage position in the middle of the sensor distribution line to obtain balanced data rates at both sides. To reach a balanced position in the central point, the data rates at the left will be built up as Σ Left and the right as Σ Right.

Σ Left will be increased when it is smaller than or equal to Σ Right, and vice versa. Σ Left (Σ Right) gets an increased value at a node when their respective data rate is not zero. Every time Σ Left and Σ Right reach your destination at positions with no data rates in-between, they are standing at nodes “*i*” and “*j*” for us to apply to output the optimal data storage node. Because the linear network is scanned at once and only counts producers and consumers, the difficulty of this algorithm is *O*(*m* + *n*) where *m* is the number of producers and *n* of consumers.

### 3.3. Genetic Algorithm

The discovery of genetic algorithms (GAs) was proposed by Goldberg. GA is a randomized global search approach that solves problems by iterative processes noted from natural evolution. Based on the search and reproduction of the fittest, GA simultaneously exploits better solutions without any preconsideration, such as continuity and unimodality. GA has been applicable for many difficult optimization problems in efficient manner when compared with other existing optimization algorithms to obtain multiple local optimum solutions.

GA is an optimization and stochastic global search process, based on the principles of genetics and natural selection. GA allows a population collected of many individuals to develop under particular selection rules to a state that maximizes the “fitness.”

Pseudocode for genetic algorithm is as follows.

- Initialization: the initial population of the solutions is generally produced randomly over the search space. On the other hand, area-specific knowledge or other information can be effortlessly built in.
- Evaluation: formerly the population is initialized once or an offspring population is produced; the fitness values of the candidate solutions are computed.
- Selection: selection assigns more copies of those solutions with higher fitness values and therefore inflicts the survival-of-the-fittest mechanism on the candidate solutions. The main thought of selection is to desire better solutions to worse ones, and many selection processes have been proposed to achieve this idea, together with roulette-wheel selection, stochastic universal selection, ranking selection, and tournament selection.
- Recombination: recombination combines component of two or more parental solutions to generate new, probably improved solutions (i.e., offspring).
- Mutation: at the same time as recombination operates on two or more parental chromosomes, mutation locally but randomly alters a solution. Yet again, there are numerous variations of mutation; however it generally involves one or more changes being made to an individual's trait or traits.
- Replacement: the offspring population formed by selection, recombination, and mutation replaces the original parental population.
- The steps (ii)–(vi) are repeated until a terminating condition is met.

### 3.4. Particle Swarm Optimization

PSO proposed by Kennedy and Eberhart can be found in [12]. PSO algorithm is annoyed by the social behavior of a group of migrating birds trying to reach to indefinite destination. Each solution is termed as “bird” in the flock and is known to as a “particle.” A particle is corresponding to a chromosome in genetic algorithms (GAs) which can be found in [13]. Not like GAs, the evolutionary procedure in the PSO does not create new birds from parent ones. Instead the birds in the population only build up their social behavior and result in their movement towards a destination which can be found in [14].

A group of birds communicate together when they fly. Each bird appears in a particular direction, and they communicate collectively and recognize the bird that is in the best location. Consequently, each bird speeds in the direction of the best bird through a velocity which is based on its current position. All birds inspect the search space from their new local location, and the process is repeated until the flock arrives at a favoured destination. It is to be observed that the procedure comprises both social interaction and intelligence, so that the birds discover their own experience called local search and also the experience of others around them called global search.

The process is initiated with a collection of random particles, *N*. The *i*th particle is denoted by its position as a point in *S*-dimensional space, where the *S* denotes the number of variables. During the process, each particle *i* observes three values, namely, its current position (*X*_*i*_), the best position it arrived at in previous cycles (*P*_*i*_), and its flying velocity (*V*_*i*_). These three values are denoted as follows:

(8) $$\begin{array}{c} \text{Current}\;\text{position}\;\;X_{i}=\left(x_{i1},x_{i2},\ldots ,x_{iS}\right),\\ \text{Best}\;\;\text{previous}\;\;\text{position}\;\;P_{i}=\left(p_{i1},p_{i2},\ldots ,p_{iS}\right),\\ \text{Flying}\;\text{velocity}\;\;V_{i}=\left(v_{i1},v_{i2},\ldots ,v_{iS}\right). \end{array}$$

In each time interval (cycle), the position (*P*_*g*_) of the best particle (*g*) is computed as the best fitness of all particles.

Thus, each particle updates its velocity *V*_*i*_ to get closer to the best particle *g*, as follows:

(9) New  Vi=ω×current  Vi+c1×rand()×Pi−Xi+c2×Rand()×Pi−Xi.

As such, using the new velocity *V*_*i*_, the particle's updated position becomes

(10) $$\begin{array}{c} \text{New}\;\text{position}\;\;X_{i}=\text{current}\;\text{position}\;\;X_{i}+\text{New}\;\;V_{i}, \end{array}$$

where *c*_1_ and *c*_2_ represent two positive constants named as learning factors (usually *c*_1_ = *c*_2_ = 2), rand() and Rand() denote two random functions in the range [0,1], *V*_max⁡_ is an upper limit on the maximum change of particle velocity which can be found in [15], and *ω* denotes an inertia weight employed as an enhancement to manage the influence of the previous history of velocities on the current velocity. The *ω* balances the global search and the local search; and it is introduced to minimize linearly with time from a value of 1.4–0.5 which can be found in [16]. For this itself global search initiates with a large weight and then decreases with time to favor local search over global search which can be found in [17, 18].

It is observed that the second term in (9) indicates cognition or the private judgment of the particle when compared with its current position to its own best position. The third term in (9) denotes the social collaboration between the particles and compares a particle's current position to that of the best particle. Furthermore, in order to control the change that occurs in the particles velocities, upper and lower bounds for velocity change are limited to a user-specified value of *V*_max⁡_. Once the new position of a particle is computed using (8), the particle, then, flies towards it. Therefore, the main parameters used in the PSO are the population size (number of birds), number of generation cycles, and the maximum change of particle velocities *V*_max⁡_ and *ω*:

(11) $$\begin{array}{c} V_{\max}\geq V_{i}\geq -V_{\max}. \end{array}$$

The random number of nodes is initialized and it is denoted by *N* or *k*. Compute a relative weight of edges *G* by initializing the value of weight factor *ω*, then calculate the fitness function for all the nodes. For each distance of the sensor node the best position is determined as a *pBest*. If *fitness*(*i*) is better than *pBest*, *pBest*(*i*) = *fitness*(*i*) function is satisfied and the execution ends and the data is stored in the nodes. If the condition is not satisfied, assign an energy cost to the fitness value and then calculate the temporary energy cost for each node. The calculated temporary energy cost and the energy cost are satisfied with each other; the algorithm ends and the data is stored successfully in the nodes for future retrieval process.

Detailed pseudocode of PSO algorithm is as follows.

(1) A population of agents is created randomly:
  (12) $$\begin{array}{c} X_{i}=\left(P_{1},P_{2},P_{3},\ldots ,P_{N}\right). \end{array}$$
(2) Each particle's position according to the objective function is evaluated. In this case it is the total operational cost given by *C* for each particle and evaluate their fitness (i.e., minimization of the objective function).
(3) Cycle = 1.
(4) Repeat.
(5) Update the velocity of the particles according to the formula
  (13) Vit=Vit−1+Ciripbestt−xit−1+C2r2gbestt−xit−1.
 
*c* = acceleration factor. *r* = random values between 1 and 0.
(6) Evaluate the velocity to ascertain if it is the range of
  (14) $$\begin{array}{c} V_{\max}\leq V_{i}\leq V_{\min}. \end{array}$$
(7) Move particles to their new position:
  (15) $$\begin{array}{c} X_{i}\left(t\right)=X_{i}\left(t-1\right)+V_{i}\left(t\right). \end{array}$$
(8) Evaluate ensuring that limits have not been exceeded.
(9) Compare the particle's fitness evaluation with its previous *pbest*. If the current value is better than the previous *pbest*, then set the *pbest* value equal to the current value and the *pbest* location equal to the current location in the *N* dimensional search space.
(10) Compare the best current fitness evaluation with the population *gbest*. If the current value is better than the population *gbest*, then reset the *gbest* to the current best position and the fitness value to current fitness value.
(11) Check if stopping criterion had been met. If not update the cycle and go back to step (5).
(12) End when the stopping criterion, which here is the number of iterations, has been met.

Begin.

Generate random population of *N* solutions (particles).

For each individual *i* ∈ *N*, calculate *fitness*(*i*).

Initialize the value of the weight factor *ω*.

For each particle, set *pBest* as the best position of particle *i*.

If *fitness*(*i*) is better than *pBest*, then *pBest*(*i*) = *fitness*(*i*).

Else set *gBest* as the best fitness of all particles.

End.

For each particle, do calculation of particle velocity according to (3).

Update particle position according to (4).

End.

Update the value of the weight factor *ω*.

Check if termination = true.

End.

### 3.5. Hybrid Genetic and Particle Swarm Optimization for Data Storage Problem

In the following section what has been primarily focused on and discussed are the proposed infrastructure and rationale of the hybrid GA-PSO. As indicated, GA and PSO simultaneously function given the very same initial population. Hence when resolving problems associated with data storage, randomly chosen individuals are deployed as part of the hybrid approach. Individuals so randomly generated may be considered GA chromosomes or PSO particles, respectively. Individual assorting is basically carried out on the basis of fitness and also with regard to top of the individuals which are fed as part of the real-coded GA so that new individuals may be created through the processes of mutation as well as crossover. Real-coded GA crossover operator is then employed through the process of borrowing both vectors linear combination concept, which basically is representative of the two individuals that are part of the proposed algorithm, and possesses a 100% crossover probability. Random mutation operator here for real-coded GA proposed individual modification by using a random number as part of the problem's domain having a probability of 20%. Hence new individuals that are formed by the real-coded GA here are then employed using PSO method to make necessary adjustments in the remaining particles.

Adjustment process of PSO method top particles basically incorporates choosing global best particle, neighbourhood best particles, and velocity updates. Here population's global best particle is ascertained as per fitness value that has been sorted. The neighbourhood best particles selection is carried out initially by equally dividing 2*N* particles and segregating them as *N* neighbourhoods and then further by assigning each particle a better fitness value for every neighbourhood as the neighbourhood best particle. Considering the equation above velocity and position updates for each of the 2*N* particles are then carried out. Sorting of the results is then carried out in lieu of groundwork for repetition of the entire run and it terminates when it satisfies a convergence criterion that is based on the standard deviation of the objective function values of *N* + 1 best individuals of the population. It is defined as follows:

(16) $$\begin{array}{c} S_{f}={\left[\overset{N+1}{\underset{i=1}{\sum }}\frac{{\left(f\left(x_{i}\right)-\overset{-}{f}\right)}^{2}}{N+1}\right]}^{1/2}<v, \end{array}$$

where $\overset{-}{f}=\sum_{i=1}^{N+1}f(x_{i})/(N+1)$ and *v* = 1 × 10^−4^.

The proposed algorithm is as follows.

(1) Initialization: generate a population *X*_*i*_, *i* = 1,2,…, *SN*.
(2) Repeat.
(3) Evaluation and ranking: evaluate the fitness of each of the individuals.
(4) GA method: apply real-coded GA operators (crossover and mutation) to the top individuals and create another individual.
  (i) Selection: from the population, select the best individuals according to fitness.
  (ii) 100% Crossover: using the best individuals, apply two-parent crossover to update the best 2*N* individuals by the following equations:
    (17) xi′=Uniform0,1xi+1−Uniform0,1xi+1            i=1,2,…,N−1,xi′=Uniform0,1xi+1−Uniform0,1xi               i=N.
  (iii) 20% mutation: apply mutation with a 20% mutation probability to the best *N* updated chromosomes according to the equation
    (18) $$\begin{array}{c} x_{i}^{\prime }=x_{k}+\text{rand}\times N\left(0,1\right). \end{array}$$
(5) PSO method: apply PSO operates (velocity and position updates) for updating the 2*N* individuals with worst fitness.
   Updates: the particles' velocities and positions are updated by the following equation:
    (19) VidNew=w×Vidold+c1×rand×pid−xidold+c2×rand×pgd−xidold,
   where *c*_1_ = *c*_2_ = 2  and  *w* = [(0.5 + rand/2.0)]. Equation (19) illustrates that the new velocity for each individual particle is updated according to its previous velocity (*V*_id_), the best location in the neighborhood about the particle (*p*_id_), and the global best location (*p*_*gd*_). A particle's velocity *V*_max⁡_ is set to certain fraction of the range of the search space in each dimension, until the termination criterion is reached.

## 4. Clustering Based on Data Storage Using FCM

There are many existing problems associated with wireless sensor's traditional data storage algorithms, namely, deficiency in terms of adaptability as well as load balancing, high energy consumption, prolonged network cycle lifetime, undue high delay access rate, and a host of others. The paper presented here primarily has proposed an adaptive clustering based on the data storage (CBDS) algorithm which is conceptually based on FCM which addresses related issues and problems. By conducting data storage method analysis we studied the process of determining data storage nodes that were categorized as limited energy of sensor networks as well as other consumers existing in the network. As a conclusion of all the research a common storage strategy was devised which basically combined local and distributed storage with sensor networks set's centralized storage. Finally, a comparison was drawn between CBDS and related algorithms. Experiments conducted have clearly shown that CBDS most certainly has numerous inherent advantages which include its ability to be self-adaptive and to balance loads, having low access latency, and consuming less energy in comparison with traditional algorithms; hence FCM may be termed as being more advantageous with respect to data storage.

## 5. Fuzzy Clustering Approach

Sensor network nodes have to be organized into various subgroups referred to as clusters. Clustering thus refers to the hierarchal structure organizing process that involves the entire sensor network which facilitates greater efficiency in terms of use of scarce resources. However, in data mining, clustering is basically a part of the exploratory data analysis that incorporates assessment of the data which is done in a random manner to identify if there is any inherent structure that exists therein. Clustering main aim is identifying natural data groupings in large data sets. Partitioned clustering involves main task of partitioning entities set into various homogeneous clusters in lieu of those that possess suitable similarity measures.

Assuming that similarity measure is considered as the Euclidian distance, in that case the partitioned clusters will become spatial neighbouring nodes required by wireless sensor networks. In literature there are varying approaches employed to acquire data partition as *N*_*c*_ clusters using hard fuzzy or possibilistic [19, 20]. Fuzzy clustering is considered more to be an objective function based method that is usually employed to create a divide in the dataset forming set of groups or clusters. Contrastingly when considering standard (crisp) clustering, fuzzy clustering provides options that allow assigning of a data point to more than a single cluster, in a manner wherein overlapping clusters may be handled suitably. This basically implies that entities are permitted to belong to more than a single cluster possessing varying membership degrees.

A prototype here represents a cluster, wherein it contains a cluster centre and information regarding cluster size and shape. Computation of the degree of membership refers to data point that a cluster belongs to and is carried out by assessing distance of the data point to the clusters centre bearing in mind cluster size and shape information. Associated practical problems possess a fuzzy nature and various fuzzy clustering methods have been developed in order to address these problems. Fuzzy clustering algorithms are basically unendorsed algorithms that are employed for data partitioning as predefined cluster numbers of clusters possessing fuzzy boundaries which are extensively used for purposes of recognition of geometrical shapes in image processing, classification, and approximation of problems. Taking into consideration application in sensor nodes WSN fuzzy clustering will facilitate it (node) to belong to different clusters that have varying degrees of membership that provide inherent support whilst overlapping clustering occurs. Overlapping clusters as such have the capability of boosting flexibility of cluster base routing protocol as opposed to node failure or compromise. This is because overlapping clusters may offer multiple paths between every overlapping cluster pair [21, 22].

*Fuzzy c-means Algorithm*. Among the fuzzy clustering methods the most widely employed one is fuzzy *c*-means (FCM) algorithm [23] as its basis conceptually is based on fuzzy *c*-partition, which was introduced by Ruspini [24]. FCM algorithm carries out an intensive search for spherical clusters. Assume that *N*_*s*_ sensor nodes are deployed in a sensor field with area *M* × *M* m^2^. Each node *n*_*i*_ where 1 ≤ *i* ≤ *N*_*s*_ must be mapped to a cluster *C*_*j*_ where 1 ≤ *j* ≤ *N*_*c*_, and *N*_*c*_ is the number of clusters, such that 2 ≤ *N*_*c*_ ≤ *N*_*s*_. Our goal is to determine the optimal number of clusters *N*_*c*_ using fuzzy clustering approach. Consider that the data matrix *Z* consists of vectors *z*_*k*_, *k* = 1,…, *N*_*s*_, contained in its column. The vectors are partitioned into *N*_*c*_ clusters, represented by prototype vectors $C_{i}^{c(l)}={\left[\begin{array}{cc} C_{i1}^{c} & C_{in}^{c} \end{array}\right]}^{T}$. The algorithm is based on the calculation of fuzzy partition matrix *U*_*F*_ under the following constraints:

(20) $$\begin{array}{c} \mu_{jk}\in \left[0,1\right],\;1\leq j\leq N_{c},\;\;1\leq k\leq N_{s},\\ \overset{N_{c}}{\underset{j=1}{\sum }}\mu_{jk}=1,\;1\leq k\leq N_{s},\\ 0<\overset{N_{c}}{\underset{j=1}{\sum }}\mu_{jk}<N_{s},\;1\leq j\leq N_{c}, \end{array}$$

where *μ*_*jk*_ is the membership value of *z*_*k*_ in cluster *i*. The minimizing criterion used to define clusters, that is, optimal fuzzy *c*-partition, is defined as

(21) $$\begin{array}{c} J_{c}=\left(Z,U_{F},C^{c}\right)=\overset{N_{s}}{\underset{k=1}{\sum }}\;\overset{N_{c}}{\underset{j=1}{\sum }}{\left(\mu_{jk}\right)}^{m_{p}}{\left\|Z_{k}-C_{j}^{c}\right\|}^{2}. \end{array}$$

‖·‖ is the Euclidean distance norm and the weighting exponent *m*_*p*_ > 1.

**Step 1 .**

Choose a value for *N*_*c*_, *m*_*p*_, and *ε*, a small positive constant. Initialize randomly a fuzzy *c*-partition *U*_*F*_^0^ satisfying (20).

For the iteration *l* = 1,2,…, compute the cluster center:

(22) $$\begin{array}{c} C_{i}^{c(l)}=\frac{\sum_{k=1}^{N_{s}}{\left[\mu_{ik}^{\left(l\right)}\right]}^{m_{p}}Z_{k}}{\sum_{k=1}^{N_{s}}{\left[\mu_{ik}^{\left(l\right)}\right]}^{m_{p}}}. \end{array}$$

**Step 2 .**

Compute the new partition matrix:

(23) $$\begin{array}{c} \mu_{ik}^{\left(l\right)}=\frac{1}{\sum_{j=1}^{N_{c}}{\left[{\left\|Z_{k}-C_{i}^{c\left(l\right)}\right\|}^{2}/{\left\|Z_{k}-C_{i}^{c\left(l\right)}\right\|}^{2}\right]}^{2/\left(m_{p}-1\right)}}. \end{array}$$

**Step 3 .**

Compare *U*_*F*_^*l*^ with *U*_*F*_^*l*−1^. If ‖*U*_*F*_^*l*^ − *U*_*F*_^*l*−1^‖ < *ε*_*t*_ or a predefined number of iterations is reached, the process ends.

Here using the testing error criteria the number of clusters has been determined enabling even distribution of clusters all across the field that has been employed and this enhances load balancing that is required for WSN.

### 5.1. Fuzzy Cluster Validity Measures

Determining and identifying a technique or method that is both excellent and effective in finding clusters in data are dependent relatively on various aspects which include data size, whether or not data matches algorithm, and algorithm parameter selection. Selection of number of clusters may be carried out in advance or can be left as being routinely ascertained while employing cluster validity measures. The various scalar validity measures with reference to fuzzy clustering include partition coefficient (PC), classification entropy (CE), partition index (PI), separation index (SI), alternative Dunn index (ADI), and Xie and Beni's (XB) index. Xie and Beni's index has been shown as better index to indicate the correct number of clusters in many practical problems [25, 26]. It aims to quantify the ratio of the total variation *σ* within clusters to the minimum separation sep of clusters as

(24) $$\begin{array}{c} \sigma \left(U_{F},C^{c},Z\right)=\overset{N_{s}}{\underset{k=1}{\sum }}\;\overset{N_{c}}{\underset{j=1}{\sum }}{\left(\mu_{jk}\right)}^{2}{\left\|Z_{k}-C_{j}^{c}\right\|}^{2}, \end{array}$$

where *Z* is the feature vector and sep(*C* ^*c*^) = min⁡_*k*≠*i*_⁡{‖*Z*_*k*_ − *C*_*j*_^*c*^‖^2^}.

The *XB* index is then given as

(25) $$\begin{array}{c} XB\left(U_{F},C^{c},Z\right)=\frac{\sigma \left(U_{F},C^{c},Z\right)}{N_{s}\text{sep}\left(C^{c}\right)}. \end{array}$$

As the partitioning is more or less compacted and is also distributed well, the value of *σ* should be low, while sep should be high. Therefore, *XB* should have a low value when data have been appropriately clustered, which means if the *XB* index for a particular tuple (*N*_*s*1_, *N*_*c*1_) is *XB*_1_ and that of other tuples (*N*_*s*2_, *N*_*c*2_) is *XB*_2_ and if *XB*_1_ < *XB*_2_, then partition corresponding to (*N*_*s*1_, *N*_*c*1_) is taken better than (*N*_*s*2_, *N*_*c*2_). The *XB* index has been found to be more able to indicate correct number of partitions in the data [19] for a wide range of the choice of numbers of clusters. Hence *XB* index is used as the criterion to get the optimized numbers of clusters.

## 6. Experimental Results

In this section the performance of proposed multiple subtables is measured on the basis of CFD. The experimental setup is considered for the one-to-one model to determine the optimal storage node *k*. There are only one producer *i* and one consumer *j* to deal with information in the network. The optimal storage cost location should be attained with minimum *C*_total_(*k*). The simulation parameters taken into consideration for this evaluation are listed in Table 1.

In order to evaluate the performance of proposed PSO algorithm with FCM clustering, a wireless sensor network is implemented in simulator to execute some experiments for the data storage position. In the simulator, sensor node and storage node are randomly deployed in a 400 × 400 square area, and the sink node is in the center.

### 6.1. Average Energy Consumption Comparison

The performance of proposed hybrid PSO-FCM clustering-based data storage strategy is compared with conventional approaches like centralized data storage (CDS), optimal data storage (ODS), near-optimal data storage (NDS), and GHT. CDS is a centralized strategy. It opts for the center node in the network area as the base station to which producers deliver data and from which consumers query data. ODS and NDS approaches are elaborately described in [8].

**Figure 4:**
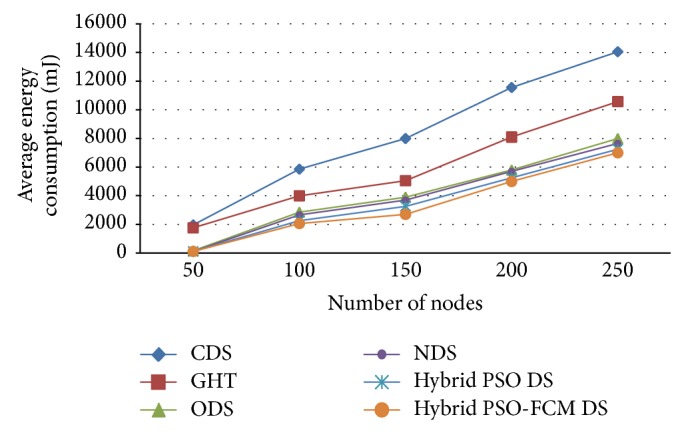
Energy consumption with number of consumers.

Figure 4 outlines the relationship that exists between energy consumed and respective consumers. Conditions applicable here are *m* = 400; when there is an increase in consumers from 50 to 650, network energy consumption will also increase; however increase in energy consumed by cluster-based data storage is lesser than the CDS, GHT, ODS, NDS, and hybrid PSO DS. Inherent difference gradually increases, which exists between both optimal and proposed algorithm and this occurs when there is an increase in storage node number and it becomes large. Finally from Figure 4 it is obvious that the proposed hybrid PSO-FCM based DS is maintaining lower energy consumption when compared with the approaches like CDS, GHT, ODS, and NDS.

### 6.2. Network Lifetime Comparison

Problems associated with clustering inherently may lead to major issues, along with the process of ascertaining the number of clusters. In a situation where other parameters have been fixed, subsequent changes while evaluating clusters would have an impact average delay, network's energy consumption, and also the life cycle. Initially, *m* = 300, *n* = 300, and the clustering process is done using proposed hybrid PSO-FCM DS. In existing process the clustering was not done. Thus the network lifetime gets increased in proposed method more than the existing methods.

**Figure 5:**
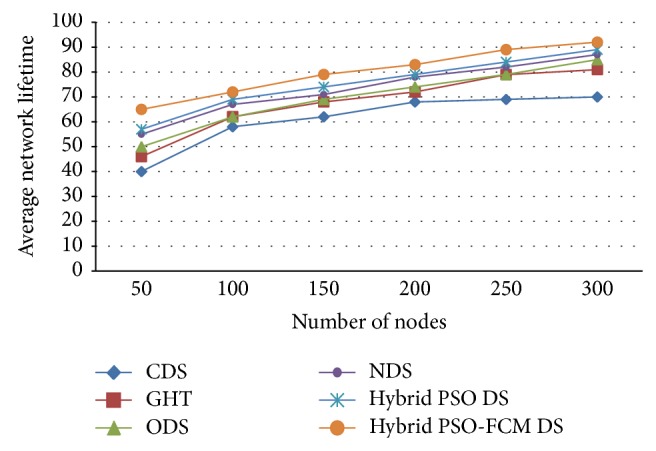
Network lifetime with different number of storage nodes.

Figure 5 here illustrates average network lifetime possessing numerous storage nodes. What is apparent here is that hybrid PSO with clustering has been proposed based on the metaheuristic algorithm that greatly impacts and affects functional aspect of the entire network's lifetime. The network's lifetime has been prolonged through the application process of FCM with hybrid PSO that has been proposed here and which is based on the metaheuristic algorithm. The metaheuristic algorithm effectively elongates network lifetime through numerous small storage nodes, bearing in mind that the aim of deploying the algorithm is minimizing overall energy cost. For instance, 10 storage nodes are considered; network lifetime increases considerably. From Figure 5 it is concluded that the proposed hybrid PSO-FCM DS has higher average network lifetime when compared to existing CDS, GHT, ODS, NDS, and hybrid PSO DS.

### 6.3. Average Run Time Comparison

In this section, the computing complexity of different algorithms is illustrated by means of comparing the run time in the same testing environment. The approaches ODS, NDS, CDS, GHT, hybrid PSO DS, and proposed hybrid PSO-FCM DS are tested in the same PC with Pentium IV 1.70 GHz CPU and 256 MB main memory.

**Figure 6:**
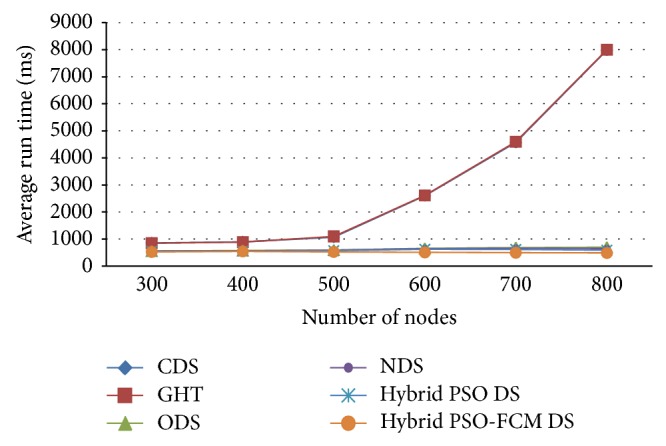
Network Lifetime with different number of storage nodes.

Figure 6 shows the average run time to get the storage position with different number of nodes when *m* = 200 and *n* = 50 in 100 simulation iterations. CDS and GHT can get the storage position directly. They have almost the same run time and their curves overlap. Since ODS needs to try and test each sensor node, its run time increases tremendously with the number of nodes in the network, which makes it infeasible in a very large-scale network. In contrast, NDS increases much mildly because it only needs to test neighbouring nodes. However, the proposed hybrid PSO-FCM DS method attains low average run time compared to the existing CDS, GHT, ODS, NDS, and hybrid PSO DS approaches.

### 6.4. Average Delay Comparison

**Figure 7:**
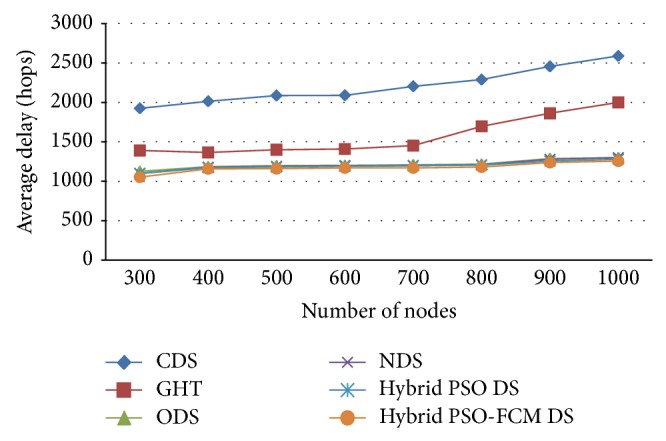
Average delay comparison.

Figure 7 entails details of the comparison drawn on the approaches average delay. Figure 7 shows the average delay for nodes between 400 and 1000 and *m* = 200 and *n* = 50 in 100 simulation iterations. Observations drawn on the basis of Figure 6 show that the proposed metaheuristic which is based on the hybrid PSO with FCM clustering approach delivers significant results that have a lesser average delay in comparison with ODS, GHT, ODS, NDS, and hybrid PSO approaches. Figure 7 indicates that ascending clusters that resulted in hybrid PSO along with the delay of FCM clustering were reduced considerably.

## 7. Conclusion and Future Work

Data storage has turned out to be a significant problem in sensor networks as a large quantity of collected data is required to be achieved for future information retrieval. This paper reflects on the storage node placement problem intended to reduce the total energy cost for collecting data to the storage nodes. This paper introduces a metaheuristic nature motivated from optimization algorithm to deal with the data storage problem in wireless sensor network. In this data storage approach, storage node has been presented to reduce the transmission of raw data and to extend the lifetime of all wireless sensor networks. In the end what has been proposed is self-adaptive clustering on the basis of the data storage algorithm which deploys FCM clustering technology.

This basically segregates network as various storage clusters wherein every cluster's storage node is adaptive in nature and makes adjustments with regard to data storage so that energy consumed may be lessened. At last a comparison was drawn out on experiments carried out on using associated algorithms and self-adaptive clustering that is based on data storage algorithm. These experiments indicated that self-adaptive clustering which is basically done on the basis of the data storage algorithm has certain inherent advantages. These include being self-adaptive, ability to balance loads, having low access latency, and also reducing energy consumption in comparison with conventional algorithms. CBDS thus can be said to be more favourable for data storage.

In order to effectively resolve storage issues, a comparison has been drawn between proposed metaheuristic algorithm and novel data storage approaches including CDS, ODS, and NDS. The hybrid proposed here that is the PSO with FCM clustering is observed to be significant as it minimizes energy consumption by choosing the storage node adaptively by taking data rates of producers, query rates of consumers, and their geographic locations into consideration. The future enhancement of this work would be to use hybrid optimization algorithm in many-to-many model.

## Figures and Tables

**Table 1:** Simulation parameters.

Parameters	Value
Number of nodes	50
Area size	400 × 400 m
Mac	802.11
Traffic source	CBR
Initial energy	100 J
Packet size (s)	40 bytes
Transmitting cost per message	0.645 mJ
Transmitting cost per byte	0.0144 mJ/byte
Receiving cost per message	0.387 mJ
Receiving cost per byte	0.00864 mJ/byte
Antenna	Omni. antenna
Radio propagation	Two-ray ground
Interface queue	Queue/drop tail
Queue length	50
Channel type	Channel/wireless channel
Routing protocol	AODV
